# Aminophosphonic Acid Functionalized Cellulose Nanofibers for Efficient Extraction of Trace Metal Ions

**DOI:** 10.3390/polym12102370

**Published:** 2020-10-15

**Authors:** Hilal Ahmad, Walaa Alharbi, Ibtisam I. BinSharfan, Rais Ahmad Khan, Ali Alsalme

**Affiliations:** 1 Division of Computational Physics, Institute for Computational Science, Ton Duc Thang University, Ho Chi Minh City 758307, Vietnam; hilalahmad@tdtu.edu.vn; 2 Faculty of Applied Sciences, Ton Duc Thang University, Ho Chi Minh City 758307, Vietnam; 3 Department of Chemistry, Faculty of Science, King Khalid University, P.O. Box 9004, Abha 62529, Saudi Arabia; Wal-harbe@kku.edu.sa; 4 Department of Chemistry, College of Science, King Saud University, Riyadh 11451, Saudi Arabia; ibtisam.i.sh@hotmail.com (I.I.B.); krais@ksu.edu.sa (R.A.K.)

**Keywords:** solid-phase extraction, toxicity, heavy metal ions, adsorption

## Abstract

Cellulose nanofibers were covalently functionalized using diethylenetriamine penta (methylene phosphonic acid) and studied for the extraction of heavy metal ions. The surface-functionalized nanofibers showed a high adsorption capacity towards heavy metal ions as compared to bare nanofibers. The elemental composition and surface morphology of the prepared bio-adsorbent was characterized by X-ray photoelectron spectroscopy, attenuated total reflectance infrared spectroscopy, field emission scanning electron microscopy, and energy dispersive spectroscopy. The prepared material was studied to develop a column-based solid phase extraction method for the preconcentration of trace metal ions and their determination by inductively coupled plasma optical emission spectroscopy. The batch experimental data was well fitted to Langmuir adsorption isotherms (R^2^ > 0.99) and follows pseudo-second-order kinetics. The experimental variables such as sample pH, equilibrium time, column breakthrough, sorption flow rate, the effect of coexisting ions, and eluent type were systematically studied and optimized accordingly. The detection limit of the proposed method was found to be 0.03, 0.05, and 0.04 µg L^−1^ for Cu(II), Pb(II), and Cd(II), respectively. Certified Reference Materials were analyzed to validate the proposed method against systematic and constant errors. At a 95% confidence level, the Student’s t-test values were less than the critical Student’s t value (4.302). The developed method was successfully employed for the preconcentration and determination of trace metal ions from real water samples such as river water and industrial effluent.

## 1. Introduction

The environmental water pollution due to the occupancy of upraised concentrations of a wide diversity of pollutants such as dyes, antibiotics, organic compounds, and heavy metal ions has been extensively reported in different parts of developed and developing nations in recent decades [1,2,3,4,5,6,7,8]. Global industrialization and the untreated discharge of wastewater into natural water sources are among the core reasons for severe water pollution [9]. Therefore, it is necessary to remove such pollutants from industrial and pharmaceutical wastewater before their discharge into the environmental water systems. At present, different water treatment technologies have been reported, such as catalysis, advanced oxidation process, chemical precipitation, electrochemical reduction, membrane separation, and adsorption [10,11,12,13,14,15,16,17,18]. However, many of them have severe limitations, such as the chemical precipitation method, which generates a large amount of toxic residue and leads to secondary pollution, while the electrochemical process is of high cost with low efficiency. Membrane separation is better and restricts the pollutants to pass into the mainstream; however, faces fouling after a particular time period [19,20]. The solid-phase extraction (SPE) technique has been considered a promising method due to its simple preparation, relatively low cost, ability to use both in batch and column procedures, faster kinetics, and high possibility to reuse the adsorbent [21,22,23].

Bio-sorbents such as cellulose, chitosan, rich husk, and modified leaves have been widely studied to remove metal ions [24,25,26,27]. In powder form, their use is restricted in column operation because of small particle size; either they agglomerate or release out from the column bed along with the sample solution. Moreover, due to limited active sites to bind with heavy metal ions, they showed less adsorption capacity. Therefore, their morphological and chemical modifications are required to enhance the adsorption performance. Bacterial cellulose (BC) is extracellular cellulose synthesized by some bacteria called *Acetobacter Xylinum*. The basic structure of BC is of the same kind as that of plant cellulose with high hydrophilicity and biodegradable [28]. BC is composed of ultrafine microfibrils with ribbon-like morphology and free from hemicellulose and lignin. Considerable research has been focused on its usage in papermaking technology, biomedicine, and the food industry [29,30,31]. BC has substantial hydroxyl groups on its backbone with the advantage of further chemical modification to improve its utility in the field of separation science [32]. The BC has been recently studied for various applications [33,34]; however, the prepared BC in the reported literature is limited in application range and adsorption capacity [35,36,37]. In this paper, we chemically functionalized the BC with diethylenetriamine penta (methylene phosphonic acid) by using the inherent surface groups of BC for the covalent functionalization of a ligand. The functionalization of BC with organic ligands enhances the adsorption properties, and the metal ions are usually adsorbed by chelation rather than the physical interaction. The nanometer dimensional network of BC fibrils causes a large surface area, and for this reason, the BC fibrils hold a sufficient amount of functionalized ligand. The phosphonic acid immobilized BC forms polynuclear complexes of the type M5L.xH2O (M = Pb(II), Cd(II) and Cu(II)), and shows a high adsorption capacity towards heavy metal ions as compare to nascent BC. This new material is highly hydrophilic due to incorporated surface groups and shows faster extraction of metal ions from aqueous media. The SPE method extracts a number of analytes over a wider pH range. It elutes the same to regenerate the column over many cycles with good reproducibility in the adsorption characteristics. SPE overcomes many drawbacks, such as using a carcinogenic organic solvent in solvent extraction, emulsion formation caused by the mutual solubility between the organic solvent and aqueous layer, and analyte loss during multi-step extraction. The incorporated surface groups of BC played a central role by enhancing the hydrophilicity and accessibility of metal ions for faster complexation. 

## 2. Materials and Methods

### 2.1. Materials

All the chemicals used were of analytical reagent grade. Bacterial cellulose nanofibers were purchased from Biocrown biotechnology (Guangzhou, China) and used after sequential rinsing with HNO_3_ (5%), acetone, and DI (deionized water). A stock solution of metal ions of 1000 ppm was procured from Sigma-Aldrich (Steinem, Germany) and used after successive dilutions. Diethylenetriamine penta (methylene phosphonic acid), dimethylformamide, and urea were procured from Sigma-Aldrich (Steinem, Germany). The buffer solutions used for the pH 2.0–2.8, 3.0–3.6, 4.0–6.0, and 7.0 were KCl-HCl, HCl-C_2_H_5_O_2_N, CH_3_COOH-CH_3_COONa, and Na_2_HPO4-C6H_8_O_7_ Merck (Mumbai, India), respectively. Certified reference material NIES 8 (vehicle exhaust particulates) and NIES 10c (rice flour) were obtained from the National Institute of Environmental Studies (Ibaraki, Japan).

### 2.2. Chemical Functionalization of Cellulose Nanofibers

**Figure 1:**
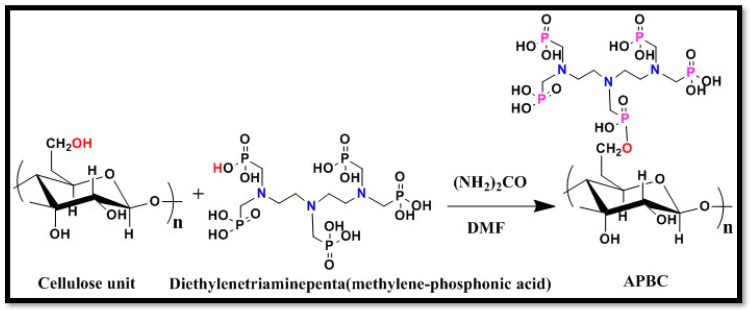
Schematic presentation of the surface modification of cellulose nanofibers.

In a typical synthesis procedure, 10 g of pretreated cellulose nanofibers was mixed with 100 mL of dimethylformamide containing 20 g of urea. After mixing for 1 h, 5 g of diethylenetriamine penta (methylene phosphonic acid) was added dropwise over 20 min. The whole reaction mixture was stirred for another 2 h at 120 °C under constant stirring. After cooling, the cellulose nanofibers were sequentially washed with the aqueous solution of propanol, 0.2 M HNO_3,_ and distilled water and dried at 60 °C in an air oven for 12 h before further use. Figure 1 shows the synthesis scheme, and the product was abbreviated as APBC nanofibers. 

### 2.3. Batch Procedure for Metal Ion Removal

In a typical batch process, 25.0 mg of APBC nanofibers adsorbent was equilibrated individually with 100 mL of 250 mg L^−1^ of Pb(II), Cd(II), and Cu(II) solution at pH 6.0 ± 0.2, in an Erlenmeyer flask (Borosil, Shanghai, China). The reaction mixture was stirred on a magnetic stirrer (Biobase, Guangzhou China) at 200 rpm and 25 °C for 12 h. An aliquot of the model solution was filtered off and analyzed to determine the residual metal ion concentration. The metal ion adsorption capacity of APBC nanofibers adsorbent in the batch test was calculated from Equation (1):

(1) $$Qe=\frac{\left(Co-Ce\right)V}{m}$$

where *Qe* is the amount of metal ions (mg) adsorbed by per gram of adsorbent, *Co* is the initial concentration of metal ions (mg L^−1^), *Ce* is the final concentration of metal ion (mg L^−1^) in the solution after adsorption, *V* is the total volume of sample solution (L) and *m* is the mass of adsorbent (g).

### 2.4. Continuous Column Procedure for Metal Ions Removal

A PTFE (poly tetra fluoro ethylene) column (Merck, Shanghai, China) (length = 15 cm and the diameter = 1 cm) fitted with the inner porous disc was used for all the column adsorption experiments. The column was packed with 200 mg of APBC adsorbent with a bed-height of 2.0 cm above the inner porous disc and was preconditioned with 5 mL of pH 6 ± 0.2 buffer solution. Each 50 mL of sample solutions containing 100 µg L^−1^ of the individual metal ion with a pH value of 6 ± 0.2 was passed through the column at an optimum flow rate of 8.0 mL min^−1^ using a peristaltic pump. After passing the complete sample solution, the column was rinsed with DI (deionized water), and the adsorbed metal ion was then desorbed with 5 mL of 1 M H_2_SO_4_ (stripping agent). The amount of the recovered metal ions in the eluent was subsequently determined by inductively coupled plasma optical emission spectrometer (ICP-OES, Model: Avio 200, Perkin Elmer, Melbourne, Australia).

### 2.5. ICP-OES Operating Conditions

Cu(II), Pb(II), and Cd(II) was determined at the axial mode of viewing plasma, using an ultrasonic nebulizer and charge-coupled detector. Other important instrumental conditions are as follows: ICP power—1.5 kW; injector—alumina injector (2.0 mm); plasma gas—Ar; plasma gas flow—8.0 L min^−1^; auxiliary gas flow—0.2 L min^−1^; nebulizer gas—0.7 L min^−1^; pressure—3.2 bar; sample uptake rate—1.5 mL min^−1^; replicates—3; integration time—10 s; wavelength (nm)—324.752 (Cu), 220.353 (Pb) and 228.802 (Cd). 

### 2.6. Material Characterization

The chemical functionalization of cellulose nanofibers surface was studied by ATR-IR (Attenuated total reflectance infrared spectroscopy) (Vertex 70v, Bruker, Ettlingen, Germany) scanned in the range of 400–4000 cm^−1^ (with the accumulation of 42 scans). The surface roughness of APBC nanofibers was studied under field emission scanning electron microscope (FESEM, JSM-7800F, JEOL, Tokyo, Japan). The elemental analysis was carried out using energy dispersive X-ray analysis spectroscopy (EDS; Bruker QUANTAX X 129eV, Berlin, Germany). The surface chemical bonding of APBC nanofibers was analyzed using X-ray photoelectron spectroscopy (XPS, Thermo Fisher Scientific ESCALAB 250Xi, Waltham, MA, USA) in a binding energy range of 0–1400 eV; using MgK alpha X-ray source at 1253.6 eV, holding detection angle of 45° with depth of 10 nm. The surface hydrophilicity was observed using a water contact angle measurement instrument (SDC-70 Shengding, China) equipped with a digital camera.

## 3. Results and Discussion

### 3.1. Characterization

**Figure 2:**
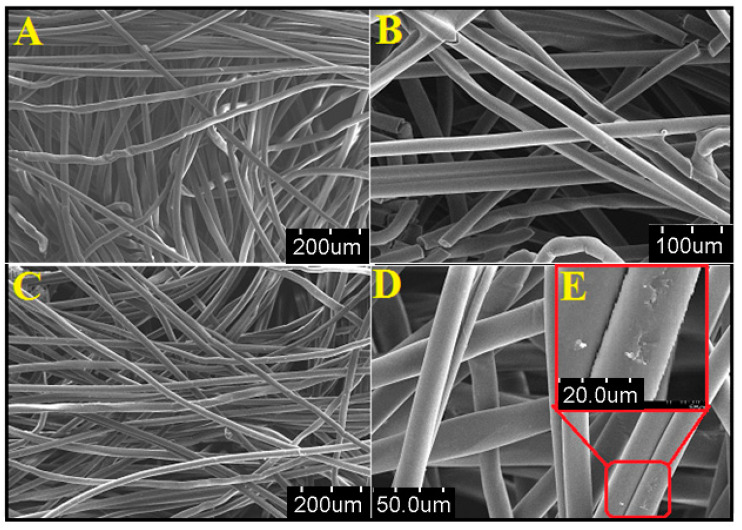
FESEM images of (**A**,**B**) nascent cellulose nanofibers at the varying resolution, (**C**,**D**) surface-modified cellulose nanofibers at the varying resolution, (**E**) inset of figure (**D**) showing surface roughness after chemical modification.

The surface morphology of nascent cellulose nanofibers and APBC adsorbent were observed under FESEM at varying magnification, as shown in Figure 2A–D. The surface texture of the nascent cellulose fibers was changed from smooth (Figure 2B) to rough after chemical modification (Figure 2C), indicates the immobilization of ligand. 

**Figure 3:**
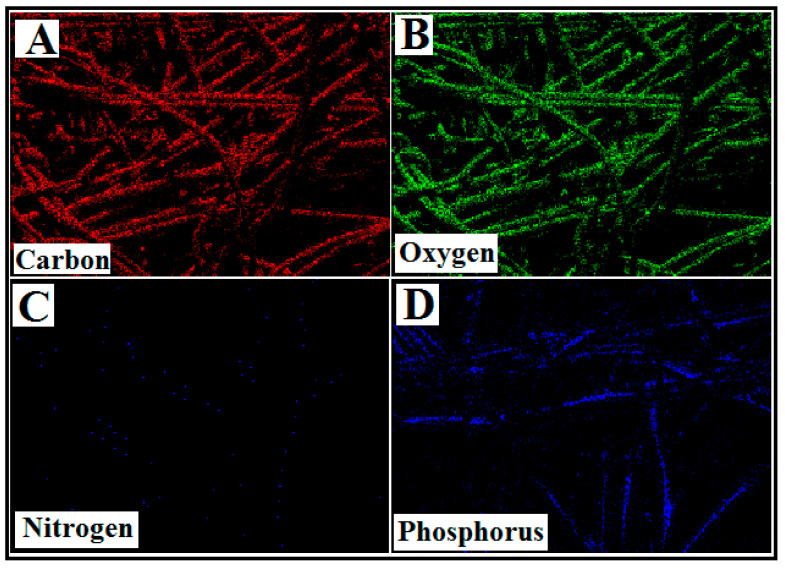
Elemental mapping of APBC surface. (**A**) Carbon map, (**B**) Oxygen map (**C**) Nitrogen map, and (**D**) Phosphorus map.

The elemental content (C, O, N, and P) of the APBC observed from the EDS spectra, and the elemental mapping of SEM images (Figure 3A–D) illustrate the homogeneous distribution of the constituent surface elements of APBC adsorbent, and the successful immobilization of poly(aminophosphonic acid) onto cellulose nanofibers. 

The XPS analyses were performed to observe the surface elements’ oxidation state and validate the incorporation of surface functionality on the cellulose nanofiber. The individual elemental details of the XPS results for APBC are given in Table 1.

**Figure 4:**
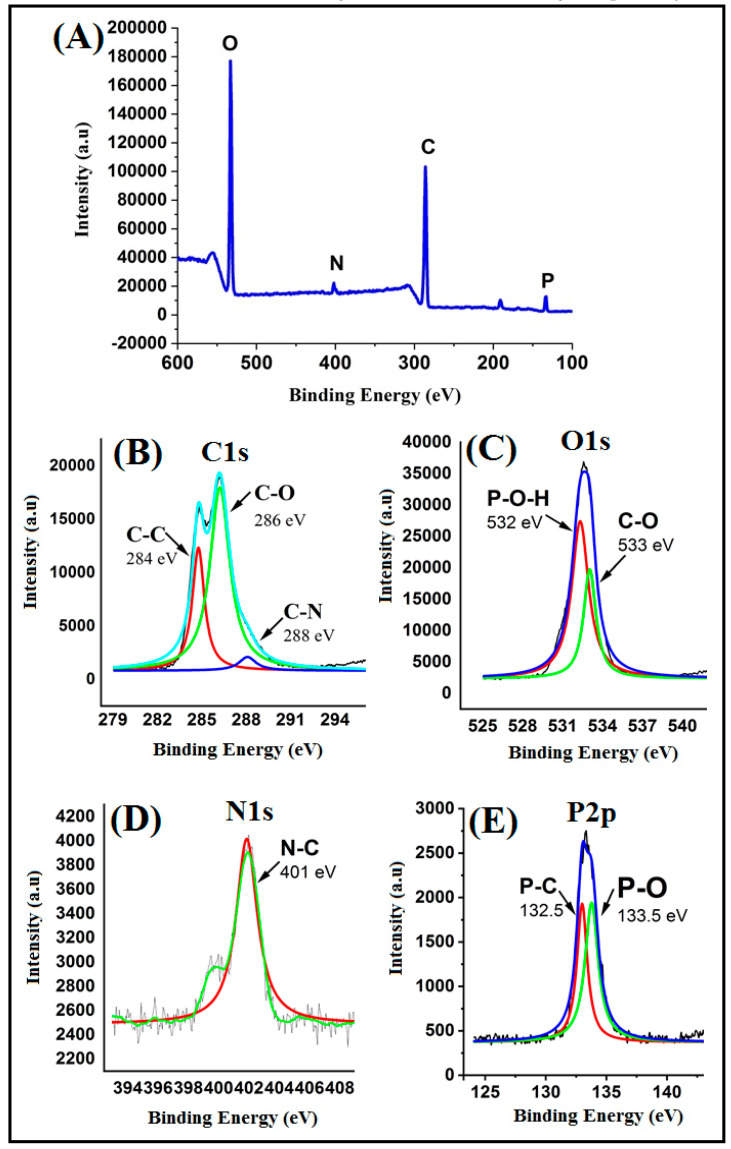
(**A**) XPS wide scan survey spectra of APBC nanofibers and deconvoluted high-resolution scans for (**B**) C1s; (**C**) O1s; (**D**) N1s and (**E**) P2p.

The wide scan spectrum of APBC adsorbent was presented in Figure 4A. The deconvoluted core energy spectra of C1s, O1s, N1s, and P2p were present in Figure 4B–E. The deconvoluted C1s spectra of APBC shows a strong C-C, C-N, and C-O peaks at 284, 288, and 286 eV, respectively, attributes to the structural carbon peaks the APBC adsorbent (Figure 4B). The deconvoluted peaks at the binding energy of 532 and 533 eV in the O1s spectrum are assigned to the P-O and C-O bonding (Figure 4C). Similarly, the deconvoluted peaks at the binding energy of 401 eV in the N1s spectrum are assigned to the N-C bonding (Figure 4D). Figure 4E shows the deconvoluted core level peaks of phosphorus (P2p) at the binding energy of 132.5 and 133.5 eV, attributes to the P-C and P-C bonding, respectively. In conclusion, all these peaks indicate the surface immobilization of poly(aminophosphonic acid) onto the cellulose nanofiber. Moreover, the results of quantitative XPS analyses are in good agreement with the EDS data of APBC nanofibers. The nitrogen gas adsorption-desorption analysis was carried out to characterize the physical properties of the adsorbent. The average surface area, pore radius, and pore volume of APBC adsorbent was calculated by the Brunauer–Emmett–Teller (BET) method and were found to be 418.15 m^2^ g^−1^, 26.22 nm, and 1.18 cm^3^ g^−1^, respectively.

**Figure 5:**
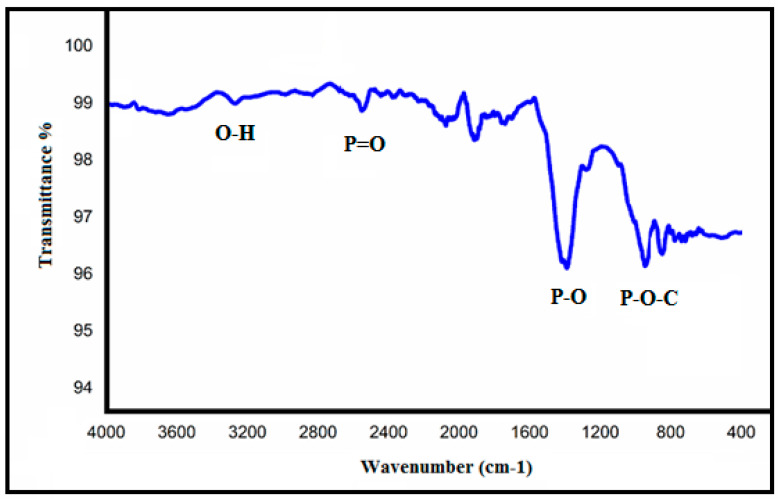
ATR-IR spectra of APBC nanofibers.

Figure 5 shows the ATR-IR spectrum of APBC adsorbent. The characteristics bands observed at 2700, 1300, and 1020 cm^−1^, attributes to the stretching vibrations of P=O double-bond, P-O bonds, and P-O-C bonds, respectively [38]. The band observed at 3300 cm^−1^ corresponds to O-H stretching vibrations of cellulose. The three main characteristic bands of the phosphonate groups ensure the successful immobilization of poly(aminophosphonic acid) onto the cellulose nanofibers surface.

### 3.2. Batch Extraction Studies

**Figure 6:**
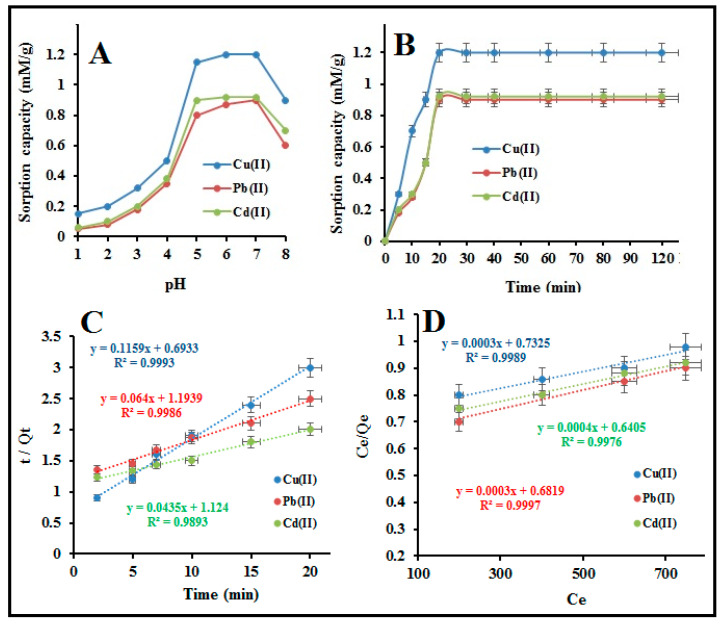
(**A**) Effect of sample pH on the sorption of metal ions, (**B**) effect of equilibrium time, (**C**) pseudo-second-order reaction kinetics, and (**D**) Langmuir isotherm models.

A series of batch extraction experiments were carried out to optimize the experimental variables like the effect of sample pH, shaking time, and adsorption isotherms. The effect of sample pH on the sorption of Pb(II), Cd(II), and Cu(II) onto APBC (modified) nanofibers sorbent was studied at a pH range of 1–8. This crucial parameter plays an important role in the uptake of metal ions since it affects both the extent of dissociation of functional groups of sorbent and the coordination sphere of metal ions in an aqueous medium [39]. To study this parameter, a 3 × 3 cm^2^ of APBC (25 mg by wt.) and nascent sorbent membrane were individually stirred with 250 mg L^−1^ of Pb(II), Cd(II), and Cu(II) solution (100 mL) for 12 h. Afterward, the sorbent was separated from the solution, and the concentration of metal ions left in the solution was determined by ICP-OES. From Figure 6A, the initial findings show high uptake of Pb(II), Cd(II), and Cu(II) by modified cellulose nanofibers, and the sorption capacity of modified nanofibers was four times higher than nascent cellulose adsorbent. The bare cellulose nanofiber was not effective in the extraction of studied metal ions at all pH values. The APBC adsorbent shows a significant increase in the extraction of studied metal ions at a pH range of 3.0–7.0.

The extraction of Pb(II), Cd(II), and Cu(II) increase as the solution pH increased from pH 1 to 5. It remained maximum at pH 6.0–7.0 due to highly favorable soft acid-soft base interaction between the metal ions and phosphonic acid groups of the sorbent [40,41]. At low pH values, the functional groups get protonated, and the surface charges become positive (-OH_2_^+^), causes weak electrostatic interaction between the sorbent and the metal ions. This results in lowering the extraction efficiency. On increasing the sample pH, the surface charge gets neutral/negative due to dissociation of functional groups (-O^−^/-OH), causes an increase in electrostatic interaction forces and ease of coordination with the metal ions, thus increases the uptake capacity. For subsequent experiments, pH 6 ± 0.2 was optimized and selected as the working pH. The effect of common alkali and alkaline earth metal and organic acids, which possibly existed with the analyte ions, has also been studied. The tolerance limit for coexisting ions were set as the maximum concentration that cause a variation of ± 5% in the emission intensity of 5 ppb of Cu(II), Pb(II) and Cd(II) concentrations. The results are presented in Table 2. It was observed that, at optimum pH, no significant decrease of analyte ions sorption was observed in presence of common existing cation, anions including humic and fulvic acids, which indicates fair selectivity of ABPC adsorbent for metal ions extraction.

The kinetics of metal-ligand equilibrium that describes the uptake rate of the metal ions is one of the important parameters that define metal ions adsorption. This crucial parameter has been studied by varying the shaking time from 5–120 min following the batch procedure. The results are presented in Figure 6B. It was observed that the adsorption kinetics was fast enough during the first 10 min. More than 50 percent of metal ions of total saturation capacity was absorbed during the first 10 min, which is necessitated for faster extraction. The adsorption equilibrium (total saturation capacity) for Cu(II), Pb(II), and Cd(II) at higher concentrations (250 ppm) was established in 15–20 min (Figure 6B). This may arise due to the strong complexation between soft acid (Cu(II), Cd(II) and Pb(II)) and soft base (phosphonic binding sites) of the functionalized nanofibers membrane. A shaking time of 20 min was optimized for the rest of the experiments. Furthermore, the kinetics data was applied to investigate the adsorption phenomenon. The mathematical equations used for the pseudo-first-order model, pseudo-second-order model, and intra-particle diffusion model are as follows [42]:

(2) ln(Q−Q(t))=lnQ−K1t

(3) $$\frac{t}{Q\left(t\right)}=\frac{1}{K_{2}Q}+\frac{t}{Q}$$

(4) $$Q\left(t\right)=K_{id}t^{1/2}+C$$

where *Q* is equilibrium value, *K1* and *K2* are coefficients variant and treated as the best-fit parameters, *K_id_* is intra-particle diffusion rate constant, and *C* is the intercept. The experimental data were well fitted to the pseudo-second-order model with the R^2^ value of 0.9939, 0.9986, and 0.9893 for Cu(II), Pb(II), and Cd(II), respectively (Figure 6C). The linearity of the pseudo-second-order model inferred the chemisorption of metal ions, suggesting that the metal ions are adsorbed through complexation rather than physical-sorption. Figure S1 shows the plots of *Q(t*) Vs. *T*_1/2_ for Cu(II), Pb(II), and Cd(II). It was observed that the intra-particle diffusion model shows multi-linearity in the adsorption of Cu(II), Pb(II), and Cd(II). This suggests the multi-step adsorption of metal ions. The first step mainly attributes to the complexation of metal ions, i.e., boundary layer diffusion. In the second step, there was a gradual adsorption of metal ions where intra-particle diffusion is the rate-determining step. In the last step, due to the lower concentration of metal ions left, the intra-particle diffusion process became slow. Similar observations were reported by Ofomaja et al. in the adsorption of metal ions onto bio-adsorbent [43].

To regenerate the APBC adsorbent for the next adsorption cycle, the metal adsorbed APBC was stirred with different mineral acids (nitric acid, hydrochloric acid, and sulfuric acid) with varying concentrations (0.5–2.0 M) and volumes (1.0–10 mL). The eluted metal ions were analyzed by ICP-OES after dilution. The obtained results are shown in Table 3. It was concluded that 5.0 mL of 1.0 M sulfuric acid is optimum for complete desorption of adsorbed metal ions. In all further studies, 5 mL of 1.0 M sulfuric acid was used as eluent.

### 3.3. Adsorption Isotherms

Adsorption isotherms are of central importance to better understand the adsorption mechanism. The adsorption equilibrium data of Cu(II), Pb(II), and Cd(II) studied at the concentration range from 200 to 750 mg L^−1^ by the batch method. They were applied for the linearized form of Langmuir, Freundlich, Tempkin, and Dubinin–Radushkevich isotherm models. In general, the Langmuir model suggests the monolayer adsorption on the surface of the adsorbent, while the surface heterogeneity of the adsorbent may be pronounced by the Freundlich model. The Temkin model demonstrated the interactions between the metal ions and the adsorbent. The Dubinin–Radushkevich (DR) isotherm was studied to interpret the sorption on a single type of uniform pores. The applicability of the model is compared by considering the correlation coefficient (R^2^) values. The linearized form of isotherm models is as follows [44,45,46].

Langmuir model equation—C_e_/Q_e_ = 1/Q_m._K_b_ + C_e_/Q_m_ (5)

Freundlich model equation—lnQ_e_ = lnk + (1/n) lnC_e_ (6)

Temkin model equation—Q_e_ = B ln a_T_ + B lnC_e_ (7)

where B = $\frac{RT}{bT}$

Dubinin-Radushkevich model—ln Qm – KE^2^ (8)

where C_e_ is the equilibrium concentration (mg L^−1^), Q_e_ is the amount adsorbed (mg g^−1^). Q_m_ is the maximum amount of metal ion adsorbed per unit weight of APBC adsorbent (mg g^−1^). K_b_ is the energy of sorption, K and n are Freundlich constants related to the adsorption capacity and adsorption intensity, respectively. T is the temperature (K), R is the universal gas constant, b_T,_ and B is the Temkin model constant.

The adsorption equilibrium data for all the studied metal ions are well fitted to the Langmuir isotherm model with the R^2^ value of 0.9989, 0.9987, and 0.9976 for Cu(II), Pb(II), and Cd(II), respectively, suggests the monolayer sorption of metal ions (Figure 6D and Figure S2a–c). Furthermore, the maximum adsorption capacity of Cu(II), Pb(II), and Cd(II) obtained from the Langmuir model at optimum pH 6.0 ± 0.2 and T = 27 ± 0.2 °C is closer to the batch equilibrium sorption capacity, shows in Table 4. The separation factor (R_L_ values) for Cu(II), Pb(II), and Cd(II) obtained from the K_b_ values (Langmuir sorption constant) is found to be 0 *<* R_L_ *<* 1), suggests that the data fit well to the Langmuir isotherm (Table 5). In conclusion, it was observed that the nature of metal ions adsorption by APBC adsorbent is mainly chemical adsorption, i.e., via metal-ligand complexation, rather than physical adsorption.

### 3.4. Hydrophilicity Test

**Figure 7:**
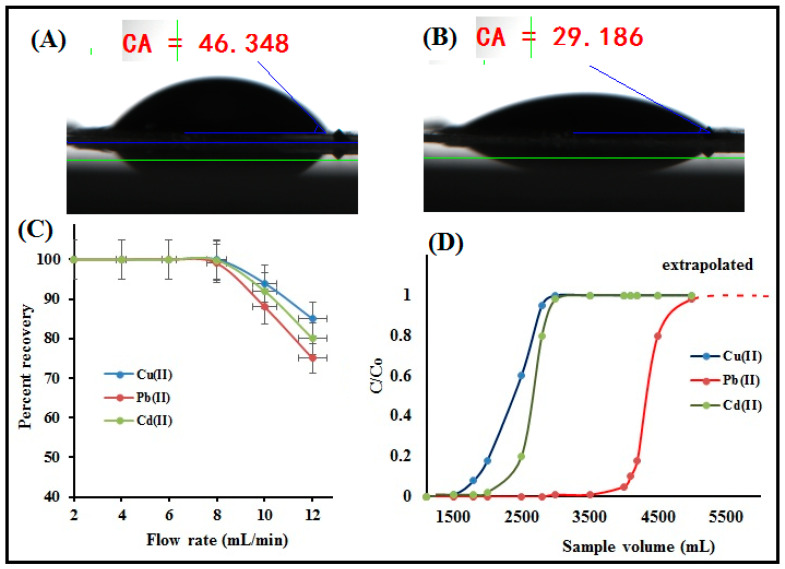
Water contact angle measurement of; (**A**) nascent cellulose nanofibers and (**B**) APBC nanofibers, (**C**) effect of column flow rate and (**D**) breakthrough curve for the extraction of Cu(II0, Pb(II) and Cd(II) (experimental conditions: sorbent amount 25.0 mg; pH = 6.0; flow rate = 8 mL min^−1^, M^n+^ = 10 mg L^−1^).

The oxygen-containing surface functional groups of APBC adsorbent led to the high hydrophilicity; thus, enhances the rate of metal ion phase transfer. To calculate the water regaining capacity, the prepared APBC composite was soaked in deionized water for 24 h, then air-dried, weighed, and dried again at 100 °C in an air oven for 24 h and weighed again. The water regaining capacity was estimated using the formula: W_t_ = (M_w_ − M_d_)/M_d_, where M_w_ was the weight of air-dried composites, and M_d_ was the weight of composites after drying at 100 °C. The water regains capacity for APBC composite was found to be 25.6 mmol g^−1^. Figure 7A,B showed that the water contact angle measurements of nascent cellulose nanofibers and surface functionalized APBC adsorbent. The data suggest the high hydrophilicity of APBC compare to nascent cellulose nanofibers, which are advantageous for column operation.

### 3.5. Effect of Sample Flow Rate

To optimize the sorption flow rate, a series of metal ion solutions (vol. 200 mL; metal ion 10 µg; pH 6.0 ± 0.2) was passed through the column at a flow range from 2 mL min^−1^ to 10 mL min^−1^. The data obtained were plotted and shown in Figure 7C. The quantitative recovery of Cu(II), Pb(II), and Cd(II) were unaffected up to a flow rate of 8 mL min^−1,^ which is comparative among column-based studies reported in previous literature. Such a high flow rate indicates the high hydrophilicity of APBC adsorbent due to the high number of surface functional groups. Above this flow rate (up to 12 mL min^−1^), the gradual decrease of 5–30 % in the recovery of metal ions were noticed. Such a decrease in adsorption at higher sample flow may due to the insufficient contact between the metal ions and the active sites of the adsorbent. Hence, a sample flow rate of 8 mL min^−1^ for all the metal ions was selected and applied for the column adsorption experiments.

### 3.6. Preconcentration and Breakthrough Studies

The direct determination of metal ions in large sample volumes is limited either due to the ultra-low level of concentrations of metal ions, which were below the instrumental detection limit, or by spectral interferences caused by coexisting ions. Preconcentration is a technique to improve the analyte concentration by transforming it from a large sample volume to a smaller one. Herein, to study the sorption of trace Cu(II), Pb(II), and Cd(II), a series of model solutions with a sample size of 1000–3000 mL contains 1.0 ug of each metal ions was passed through the column following under optimum conditions. The sorbed metal ions were then eluted, and the concentration of metal ions was subsequently determined by ICP-OES. The results are shown in Table 6. Conclusively, the quantitative recovery of Cu(II) was achieved up to a sample volume of 2900 mL while the Pb(II) and Cd(II) was quantitatively recovered up to a sample volume of 2700 mL. This could be due to the high affinity of phosphonic acids group towards Cu(II) compare to Pb(II) and Cd(II) [47,48]. At higher sample volumes (3000 mL), the percent recovery decreases (85–90%) for all the studied metals ions (Table 6). Thereby, a high preconcentration factor of 580 for Cu(II) and 540 for Pb(II) and Cd(II) was obtained. Such a high preconcentration factor is due to the high number of surface hydrophilic groups of the adsorbent and the accessible chelating sites. The corresponding preconcentration limit for Cu(II) and Pb(II)/Cd(II) was found to be 0.34 and 0.37 µg L^−1^, respectively.

To study the breakthrough curve, a 5000 mL of sample volume each contains 10 mg L^−1^ of each metal ions was passed through the column under optimum conditions. The fractions of effluent were collected at certain time intervals and analyzed by ICP-OES. Figure 7D shows the breakthrough curves for analyte ions, and the breakthrough volumes for Cu(II), Pb(II), and Cd(II) at which the concentration of the analyte is about 4–5% of initial metal concentration were found to be 1800, 4200 and 2500 mL, respectively. The breakthrough capacities obtained is very close to the batch adsorption capacity (Table 4), suggesting the potential application of APBC adsorbent for continuous column operation.

### 3.7. Analytical Figure of Merit and Real Sample Analysis

According to the IUPAC definition [49], the detection limit (LOD) obtained as the concentration of analyte ions equivalent to three times the standard deviation of mean blank signal (3*S*d; n = 11), was found as 0.03, 0.05, and 0.04 µg L^−1^ for Cu(II), Pb(II) and Cd(II), respectively. Under optimized experimental conditions, the precision of the method, evaluated in terms of relative standard deviation (RSD) for ten replicate samples contains 5 µg L^−1^ of all analyte ions, were found in the range of 2.8% and 3.5%. The recovery percent for each analyzed Cu(II), Pb(II), and Cd(II) were satisfactory and ranging from 96% to 100%. The calibration plot sketched after preconcentrating metal ion standards of concentration ranging from 0.2–100 µg L^−1^, was found linear with acceptable regression coefficient of 0.9998 for Cu(II) (A = 17.5908 X_Cu_ + 3.1250), 0.9989 for Pb(II) (A = 107.4578 X_Pb_ + 4.7348) and 0.9996 for Cd(II) (A = 9.7639 X_Cd_ + 4.6254). The limit of quantification (LOQ), calculated as 10*S*d (n = 11), was found to be 0.10, 0.16, and 0.13 µg L^−1^ for Cu(II), Pb(II), and Cd(II), respectively. The proposed method was validated by analyzing two certified reference materials (NIES 8 and NIES 10c). The measured values are in good agreement with the legislation values, as shown in Table 7. Also, the spiking analysis was carried out on two different environmental water samples (Table 7). The spiked amount of metal ions were satisfactorily recovered with a 95% confidence limit, and the mean percentage recoveries range from 96.7% to 102%, with a relative standard deviation (RSD) of less than 5%. This suggests the accuracy of the method, and the APBC packed column could be used to preconcentrate the trace metal ions in real samples for accurate determination.

## 4. Conclusions

The fine cellulose nanofibers were chemically modified using diethylenetriamine penta (methylene phosphonic acid) and systematically characterized by FESEM, XPS, BET, and ATR-IR to study the morphology and surface functional groups. The prepared material was successfully employed for the separation and preconcentration of heavy metal ions from real water samples. The abundant surface phosphonic groups are highly active to form chelates with metal ions and led to high adsorption capacity. The high hydrophilicity of APBC adsorbent makes the material suitable to use in the column procedure. The batch adsorption data were well fitted to the Langmuir isotherm model and followed pseudo-second-order kinetics. The batch adsorption capacities were close to breakthrough capacities and were found to be 76.3, 180.3, and 103.4 mg g^−1^ for Cu(II), Pb(II), and Cd(II), respectively. The proposed SPE method shows high preconcentration factors in the range 580–540 and low detection limits (0.03–0.05 µg L^−1^) for trace Cu(II), Pb(II), and Cd(II) determinations. Previous reports based on the use of modified cellulose nanofiber in the adsorption of metal ions had been compared and were summarized in Table 8. It was observed that the proposed methodology is simple with better/comparable adsorption capacities over other reported adsorbents. The method was successfully validated by analyzing certified reference materials (NIES 8 and NIES 10c) and employed to determine the trace metal contaminants in environmental water samples.

## Figures and Tables

**Table 1:** XPS data of APBC adsorbent.

Element	Peak Position (eV)	Height Cps	Fwhm (eV)	Area (p) Cps. eV	Atomic %
C1s	286.28	16713.77	1.40	25314.38	27.51
O1s	532.64	33598.79	1.92	76854.39	34.54
N1s	401.93	1497.11	1.56	3777.23	2.65
P2p	133.33	2243.30	1.90	4811.04	3.52

**Table 2:** Interference studies on the adsorption of analyte ions (experimental conditions: M^n+^ = 100 µg L^−1^, sample volume = 100 mL, pH = 6.0, flow rate 8 mL min^−1^, eluent 5 mL of 1 M sulfuric acid; N = 3).

Co-Existing Ions	Salt Added	Amount Added (×10^3^ µg L^−1^)	Recovery % (RSD)		
			Cu(II)	Pb(II)	Cd(II)
Na^+^	NaCl	5800	99.9 (3.15)	98.9 (3.75)	99.1 (3.82)
K^+^	KCl	5400	99.2 (3.65)	99.7 (3.90)	99.5 (3.65)
Ca^2+^	CaCl_2_	750	99.8(4.28)	99.5 (3.05)	99.0 (3.88)
Mg^2+^	MgCl_2_	1200	98.6 (4.34)	99.5 (4.76)	99.0 (3.94)
Cl^−^	NaCl	9800	99.9 (3.16)	99.6 (4.72)	99.6 (4.16)
Br^−^	NaBr	8200	98.5 (4.14)	99.8 (4.19)	99.6 (3.44)
CO_3_^2−^	Na_2_CO_3_	3400	97.6 (3.56)	98.5 (4.05)	98.2 (3.99)
SO_4_^2−^	Na_2_SO_4_	2200	97.5 (3.15)	97.5 (4.32)	98.5 (3.34)
NO_3_^−^	NaNO_3_	3000	99.4 (3.05)	98.3 (4.65)	99.6 (5.05)
CH_3_COO^−^	CH_3_COONa	320	98.7 (4.96)	99.5 (4.05)	99.2 (3.96)
C_6_H_5_O_7_^3−^	Na_3_C_6_H_5_O_7_	2600	98.2 (3.15)	97.5 (4.42)	98.7 (4.34)
Humic acid	-	25	97.3 (4.54)	96.8 (4.24)	96.6 (4.04)
Fulvic acid	-	25	98.4 (4.05)	97.5 (3.85)	97.5 (4.05)

**Table 3:** Effect of stripping agent on the elution of sorbed metal ions (experimental conditions: adsorbent amount = 25 mg, M^n+^ = 20 mg L^−1^, sample volume = 50 mL, pH = 6.0, shaking time = 15 min).

Eluent	Concentration	Volume (mL)	Recovery Percent		
			Cu(II)	Pb(II)	Cd(II)
HNO_3_	0.5 M	3	48	40	35
		5	75	78	73
		10	85	87	85
	1 M	3	68	72	70
		5	80	82	85
		10	95	100	98
	2 M	3	96	98	95
		5	100	99	100
		10	100	100	100
HCl	0.5 M	3	48	42	40
		5	75	75	73
		10	92	90	87
	1 M	3	65	70	66
		5	80	78	80
		10	98	99	99
	2 M	3	94	95	92
		5	98	99	100
		10	100	100	100
H_2_SO_4_	0.5 M	3	75	78	74
		5	88	88	87
		10	95	96	96
	1 M	3	92	94	96
		5	100	100	100
		10	100	100	100
	2 M	3	97	99	100
		5	100	100	100
		10	100	100	100

**Table 4:** Metal ions adsorption capacity of APBC by batch and continuous column methods.

Metal Ions	Adsorption Capacities (mg g^−1^)			Breakthrough Volume (mL)
	Batch Adsorption	Langmuir Model	Breakthrough Capacity	
Cu(II)	76.20	81.40	74.10	1800
Pb(II)	180.26	215.64	168.50	4200
Cd(II)	103.40	110.18	101.06	2500

**Table 5:** R_L_ values for Cu(II), Pb(II), and Cd(II) adsorption obtained from the Langmuir model.

C_o_		200	300	450	500	550	600	650	700	750
R_L_	Cu(II)	0.121	0.116	0.095	0.089	0.085	0.079	0.075	0.067	0.058
	Pb(II)	0.126	0.115	0.104	0.098	0.088	0.078	0.071	0.053	0.048
	Cd(II)	0.213	0.126	0.113	0.099	0.092	0.086	0.080	0.075	0.072

C_o_—Initial metal ions concentration (mg L^−1^).

**Table 6:** Preconcentration studies data for APBC packed column (column parameters: M^n+^ = 1.0 µg, pH 6, flow rate 8 mL min^−1^, eluent 5 mL of 1M H_2_SO_4_, sorbent amount 25 mg).

Sample Volume (mL)	Analyte Concentration (µg L^−1^)	Preconcentration Limit			Preconcentration Factor		
		Cu(II)	Pb(II)	Cd(II)	Cu(II)	Pb(II)	Cd(II)
1000	1.0	1.0	1.0	1.0	200	200	200
1500	0.66	0.66	0.66	0.66	300	300	300
2000	0.50	0.50	0.50	0.50	400	400	400
2500	0.40	0.40	0.40	0.40	500	500	500
2700	0.37	0.37	0.37	0.37	540	540	540
2900	0.34	0.34	-	-	580	-	-
3000	0.33	-	-	-	-	-	-

**Table 7:** Validation of the proposed methodology by analyzing Certified reference material (CRMs) and real water samples after column preconcentration (column conditions: sample volume 100 mL, flow rate 8 mL min^−1^, eluent 5 mL H_2_SO_4_).

SRMs	Analyte	Certified Values (µg g^−1^)		Found (µg g^−1^) ± sd*^a^*	*t*-Test Values*^b^*
NIES 8	Cu(II)	67		65.8 ± 0.56	1.28
	Pb(II)	219		217 ± 1.25	2.79
NIES 10c	Cd(II)	1.82		1.76 ± 0.28	0.85
	Cu(II)	4.1		3.92 ± 0.46	0.95
**Samples**	**Analyte**	**Amount Spiked (µg)**	**Found (µg L^−1^) ± sd *^a^***	**Recovery Percent (RSD) *^c^***	***t*-Test Values *^b^***
Industrial wastewater	Cu(II)	0	18.8 ± 1.42	-	
		3	21.7 ± 1.67	96.7 (0.17)	0.85
		5	23.9 ± 2.03	102 (0.25)	1.13
	Pb(II)	0	7.30 ± 0.85	-	
		3	10.25 ± 1.04	98.3 (0.26)	0.65
		5	12.40 ± 1.21	102 (0.19)	0.97
	Cd(II)	0	3.57 ± 0.56	-	
		3	6.56 ± 0.84	99.7 ± (0.18)	1.12
		5	8.57 ± 0.89	100 ± (0.15)	0.89
River water	Cu(II)	0	8.76 ± 0.91	-	
		3	11.72 ± 1.15	98.6 ± (0.21)	1.53
		5	13.75 ± 1.28	99.8 ± (1.17)	1.72
	Pb(II)	0	4.96 ±0.85	-	
		3	7.90 ± 1.65	98.0 ± (1.12)	0.98
		5	10.02 ± 1.58	101 ± (1.27)	1.17
	Cd(II)	0	3.98 ± 0.74	-	
		3	7.05 ± 1.01	102 ± (1.25)	1.28
		5	8.97 ± 1.12	99.8 ± (1.15)	1.14

a Standard deviation, N = 3; b At 95% confidence level, c relative standard deviation.

**Table 8:** Metal ions adsorption onto different cellulose fibers-based adsorbents.

Adsorbent Material	Metal Ions	Adsorption Capacity (mg g^−1^)	References
APBC	Cd, Cu, Pb	76, 108, 103	this work
BCM@APTES-EDTA	Sr	44.86	[50]
Cell-EDTA and Cell-CM	Pb, Cd	41.2, 33.2 and 63.4, 23.0	[51]
PEI-BC	Cu, Pb	148, 141	[52]
BC/PVA/GO/APT	Cu, Pb	150, 217	[53]
SP-(TA-APTES)	Cd	22.66	[54]
pBC	Cr, Cu, Re	321, 256,153	[55]

## Supplementary Materials

The following are available online at https://www.mdpi.com/2073-4360/12/10/2370/s1, Figure S1: Temkin isotherm model for the adsorption of metal ions onto APBC adsorbent, Figure S2: (a–c) intra-particle diffusion plots for Cu(II) Pb(II) and Cd(II) at 323 K; (d) D-R model for APBC adsorbent.
