# Supplementary material for: Aminophosphonic Acid Functionalized Cellulose Nanofibers for Efficient Extraction of Trace Metal Ions
Source: Polymers (Basel). 2020 Oct 15;12(10):2370. doi: 10.3390/polym12102370 (PMC7650783; doi:10.3390/polym12102370)
Supplement: Supplementary file 1 [file polymers-12-02370-s001.zip › polymers-964471-supplementary.docx]

*Supplementary Materials*

**Aminophosphonic Acid Functionalized Cellulose Nanofibers for Efficient Extraction of Trace Metal Ions**

**Hilal Ahmad ^1,2^, Walaa Alharbi ^3^, Ibtisam I. BinSharfan ^4^, Rais Ahmad Khan ^4^ and Ali Alsalme ^4,^***

^1^ Division of Computational Physics, Institute for Computational Science, Ton Duc Thang University, Ho Chi Minh City, Vietnam; hilalahmad@tdtu.edu.vn

^2^ Faculty of Applied Sciences, Ton Duc Thang University, Ho Chi Minh City, Vietnam.

^3^ Department of Chemistry, Faculty of Science, King Khalid University, P.O. Box 9004, Abha-62529, Saudi Arabia; Wal-harbe@kku.edu.sa

^4^ Department of Chemistry, College of Science, King Saud University, Riyadh-11451, Saudi Arabia; ibtisam.i.sh@hotmail.com (I.I.B.); ibtisam.i.sh@hotmail.com (R.A.K.)

***** Correspondence: aalsalme@ksu.edu.sa

**Figure S1.** Temkin isotherm model for the adsorption of metal ions onto APBC adsorbent.


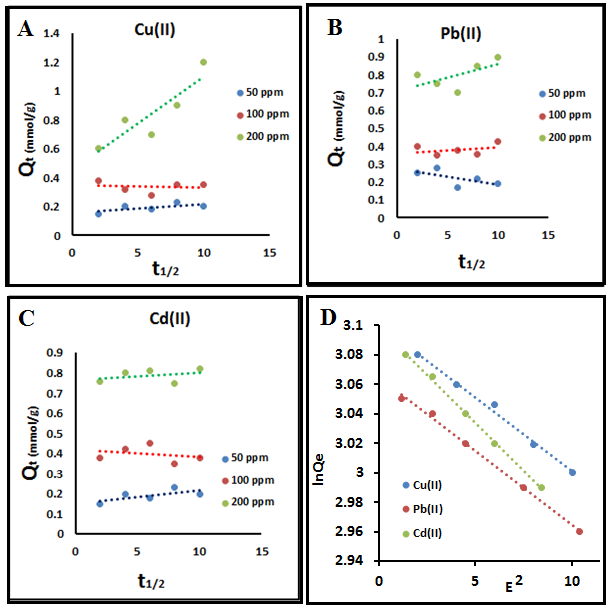


**Figure S2.** (**a**–**c**) Intra-particle diffusion plots for Cu(II) Pb(II) and Cd(II) at 323 K; (**d**) D-R model for APBC adsorbent.
